# Supplementary material for: Breath Volatile Organic Compounds in Surveillance of Gastric Cancer Patients following Radical Surgical Management
Source: Diagnostics (Basel). 2023 May 9;13(10):1670. doi: 10.3390/diagnostics13101670 (PMC10216977; doi:10.3390/diagnostics13101670)
Supplement: Supplementary file 1 [file diagnostics-13-01670-s001.zip › diagnostics-2283810-supplementary.pdf]

Supplementary information

Breath volatile organic compounds in surveillance of gastric cancer patients following radical surgical management.

Roberts Skapars[1,2], Evita Gasenko[1,2], Yoav Y. Broza[3], Armands Sivins[1,2], Inese Polaka[1], Inga Bogdanova[1,2], Andrejs Pcolkins[1,2], Viktors Veliks[1], Valdis Folkmanis[1], Anna Lescinska[1,2], Inta Liepniece-Karele[1,2], Hossam Haick[3], Ingrida Rumba-Rozenfelde[1], Marcis Leja[1,2]

[1]Institute of Clinical and Preventive Medicine, Faculty of Medicine, University of Latvia, LV-1586 Riga, Latvia

[2]Department of Abdominal and Soft Tissue Surgery, Oncology Center of Latvia, Riga East University Hospital, LV-1038 Riga, Latvia

[3]Department of Chemical Engineering and Russel Berrie Nanotechnology Institute, Technion—Israel Institute of Technology, Haifa 3200003, Israel

* Correspondence: skaparam@gmail.com (R.Š.); marcis.leja@lu.lv (M.L.)

1.1. Sampling, Preparation, and Storage of Breath Samples

Exhaled breath samples were collected in the same environmental conditions and used the same sampling process with all the volunteers, *see* details below. Room air impurity was purified from the inhaled air by a lung washout, 3 min of inhalation through a mouthpiece with a filter cartridge on the inspiratory port mouthpiece (purchased from Eco Medics, Duerten, Switzerland) diminish the exogenous VOCs concentration.[1, 2] Room samples were collected on the morning of each sampling day to identify the hospital contaminants. After the washout process, the volunteers inhaled to full lung capacity and exhaled slowly through the mouthpiece into a separate exhalation port against 10–15 cm H_2O pressure. This provides the closure of the vellum to prevent contamination through nasal entrainment. Exhaled breath consists of respiratory dead space air that is exhaled first and filled into a designated dead space bag that is then removed, followed by the alveolar air from the lungs. The alveolar breath was filled into a 1-liter Tedlar®PVF collecting bag, produced in the USA. The process of filling the Tedlar bag is a single-step process. Two bags were collected per patient for the analysis with the nanomaterial-based sensors and with the GC-MS – *see* below. Following the breath sampling, the VOCs in the breath samples were trapped and pre-concentrated in two-bed ORBO™ 420 Tenax® TA sorption tubes for gas and vapor sampling (specially treated; 35/60 mesh; 100/50 mg; purchased from Sigma-Aldrich, China) by pumping the breath content in the bag through a sorbent tube for 10 min (flow rate: 100 ml/min.). Furthermore, pumping ambient air in the collection room through to a sorbent tube for 10 min, at a rate of 100 ml/min was done to sample hospital room contaminants. To note, Tenax TA is not sensitive to humidity, it showed low H_2O trapping [3]. It was important to emphasize this since exhaled breath is assembled mainly of nitrogen, oxygen, carbon dioxide, water vapor, and inert gases [4-6] so the exposure of the high and varying room air and breath samples humidity is insignificant. The humidity levels of the samples obtained from ORBO™ 420 Tenax® were separately tested by GC-MS and showed negligible levels. The VOCs that are produced

endogenously by the biochemical processes are present in much lower amounts in exhaled breath, and many diseases are manifested through very gentle and small changes in levels of these breath VOCs.[7-10] Breakthrough should be considered when 1 liter of a breath sample is pumped through Tenax traps. Breakthrough depends on the amount of the adsorbent's material and substance in hand. The two-bed ORBO™ 420 Tenax® TA sorption tubes were created in two beds as a backup to handle breakthrough. Most VOCs should not be influenced by breakthroughs since breakthrough volumes for Tenax TA at the given conditions were more than 26 liters per gram of resin, according to the information of Sigma-Aldrich, China.

The disposable Tenax tubes were kept under refrigeration at 4 °C, until they were transferred (under refrigeration) to the laboratory amenities for the breath analysis (Laboratory for Nanomaterial-Based Devices, Technion - IIT, Israel). Six months was the maximal duration between the collection process and the analysis of the samples. We have confirmed that several VOCs that were found in the breath of lung cancer patients (decane, benzene, aldehydes and branched aldehydes) can be trapped and stored in the ORBO™ 420 Tenax® TA sorption tubes for at least half a year when the samples are refrigerated and cooled properly.

1.2. Breath Analysis with Gas-Chromatography/Mass-Spectrometry (GC-MS)

VOCs were identified by using GCMS-QP2010 instrument (Shimadzu Corporations, Japan) with an SLB-5ms capillary column (with 5% phenyl methyl siloxane; 30 m length; 0.25 mm internal diameter; 0.5 μm thicknesses (from Sigma-Aldrich, Rehovot, Israel), joint with a thermal desorption (TD) system (TD20; Shimadzu Corporation, Japan). Immediately former to the analysis, the breath VOCs were transferred from the ORBO™ 420 Tenax® TA sorption tubes to empty glass TD tubes (from Sigma-Aldrich) that are compatible with the TD system. The transfer was carried out in a glove box in a dry N_2 atmosphere and at constant temperature (T = 21°C). The TD tube undergoes a heat treatment (270 °C) in an auto-sampler thermal desorption system (TD20; Shimadzu Corporation, Japan) and the desorbed sample is temporarily stored in a stainless-steel column (150 °C); The filled TD tubes were injected into the GC-system in splitless mode at 30 cm/sec constant linear speed and under 0.70 ml/min column flow. The following oven temperature profile was set: (a) 10 min at 35 °C; (b) 4 °C/min ramp until 150 °C; (c) 10 °C/min ramp until 300 °C; and (d)15 min at 300 °C. The GC-MS chromatograms were analyzed using Mass Hunter qualitative (version B.07.00) followed by Mass Hunter quantitative analysis (version B.07.01; Agilent Technologies, USA) analysis. The compounds were tentatively identified through spectral library match NISTL.14 (National Institute of Standards and Technology, USA). Measuring external standards (2- propenenitrile, furfural, 2-butoxy ethanol, hexadecane, 4-ethyl octane, 1,2,3 trimethylbenzene, alpha methylstyrene and 2-butanone (all purchased from Sigma- Aldrich, Israel)

was done to confirm the identity and the quantity of the compounds [11]. The gaseous standards were derived using a commercial permeation/diffusion tube dilution (PDTD) system (Umwelttechnik MCZ, Germany). The carrier gas used is the purified dry nitrogen (99.9999%) from a commercial nitrogen generator (N-30, On Site Gas Systems, USA) equipped with a nitrogen purifier. The PDTD system used a temperature-controlled oven to mix a constant flow (200 ± 1 cm^3/min) of purified nitrogen with a constant mass flow of vaporized VOC(s) exiting a diffusion tube (Dynacal, VICI Metronics). To reach the desired levels and concentrations in the range from single ppb_v to several ppm_v, the nitrogen/VOC mixture exiting the PDTD system was diluted again with N_2. The VOC concentration was determined by controlling the mass flow rate of the vaporized VOC(s) through controlling the temperature of the diffusing tube, and the total volumetric nitrogen flow rate. One liter of each calibration gas mixture was pumped through an ORBO™ 420 Tenax® TA sorption tube at a rate of 100 ml/min. The calibration samples were analyzed under the same experimental conditions as the breath samples.

1.3. Breath Analysis with Nanoarray

Breath samples were analyzed using a sensor array containing eight sensors based on two types of nanomaterial: (i) organically stabilized spherical gold nanoparticles (GNPs, core diameter: 3-4 nm), and (ii) single-walled carbon nanotubes (SWCNTs). Six different organic functionalities of the GNP sensors and two for the SWCNT contributed to the chemical diversity of the sensors. The organic ligands of the GNPs provided broadly cross-reactive absorption sites for the breath VOCs.[1, 2, 7, 12]

Synthesis of the GNPs is described in refs.[2, 7, 13-15] and was achieved by drop-casting the solution onto semi-circular microelectronic transducers to form chemiresistive layers until reaching a resistance of several MΩ followed by two hours drying at ambient temperature and then baking overnight at 50 °C in a vacuum oven. The microelectronic transducers consisted of ten pairs of circular interdigitated (ID) gold electrodes on silicon with 300 nm thermal oxide (Silicon Quest International, Nevada, US). The outer diameter of the circular electrode area was 3mm, and the gap between two adjacent electrodes and the width of each electrode were both 20 □m. The SWCNT sensors were based on electrically continuous random networks of SWCNTs that were formed by drop-casting a solution of SWCNTs (from ARRY International LTD, Germany; ~30% metallic, ~70% semiconducting, average diameter = 1.5 nm, length = 7 mm) in dimethylformamide (DMF, from Sigma Aldrich Ltd., >98% purity) onto the pre-prepared electrical transducers. To accelerate the self-assembly of the SWCNTs and to evaporate the solvent after the deposition, the devices were slowly dried overnight under ambient conditions. Repetition of the procedure was done until a resistance of 100 KΩ to 10 MΩ was obtained. The microelectronic transducers for the SWCNT sensors consisted of ten pairs of 4.5 mm wide,

interdigitated Ti/Pd electrodes on silicon with two microns of thermal oxide (Silicon Quest International, Nevada, US). The gap between two adjacent electrodes was 100 □m. The SWCNT sensors were organically functionalized with cap-layers that were composed of two Polycyclic Aromatic Hydrocarbon (PAH) derivatives.[16, 17].

The sensors were mounted on a custom polytetrafluoroethylene (PTFE) circuit board located inside a stainless steel exposure chamber with a volume of 330 cm^3. While the chamber was under vacuum (~30 mtorr), the TD tube underwent a heat treatment of 250 °C in an auto-sampler thermal desorption system (TD20; Shimadzu Corporation, Japan), and the desorbed sample was temporarily stored in a stainless steel column (150 °C). When a one-way valve connecting the chamber to the column was open, the sample was sucked into the chamber, while the remaining volume was filled with N_2 until reaching atmospheric pressure in the chamber. A Keithley data logger device (model 2701 DMM) was used to sequentially acquire resistance readings from the sensor array, during the entire experiment. The whole system was controlled by a custom-made LabView program. In a typical experiment, signals from the sensor array elements were collected for 5 minutes in a vacuum, followed by 5 minutes of breath sample that filled the chamber, followed by another 5 minutes of vacuum. The GNP and SWCNT/PAH or SWCNT/HBC sensors used in this study responded rapidly and reversibly when exposed to typical VOCs in the breath [16-18].

Four sensing features were read out and extracted from the time-dependent resistance response of each sensor that related to the normalized resistance change ($(R_X-R_0)/R_0$) at the beginning of the exposure, at the middle of the exposure and at the end of the exposure (concerning the value of sensors resistance in vacuum before the exposure), and to the area beneath the time-dependent resistance response.

Each sensor responded to all (or to a certain subset) of the VOCs found in the exhaled breath samples. Breath patterns were obtained from the collective response of the sensors by applying Discriminant Factor Analysis (DFA). DFA is a linear, supervised pattern recognition method that effectively reduces the multidimensional experimental data, in which the classes to be discriminated are defined before the analysis is performed. DFA was also used as a heuristic to select the sensors with the most relevant organic functionality out of the repertoire of twenty-one, by filtering out non-contributing sensors. Important to note that our chamber included forty sensors, nineteen of them were excluded before analysis because of their "noise" response to the breath samples. The reason for selecting a certain set of sensing features for a particular problem was directly derived from their ability to discriminate between the various classification groups. DFA determines the linear combinations of the input variables (features were extracted from each sensor) so the variance in each class is minimized and the variance between classes is maximized.

The DFA output variables (i.e. canonical variables - CV) are achieved in mutually orthogonal dimensions; the first CV is the most powerful discriminating dimension.

1. Peng, G.; Hakim, M.; Broza, Y. Y.; Billan, S.; Abdah-Bortnyak, R.; Kuten, A.; Tisch, U.; Haick, H., Detection of lung, breast, colorectal, and prostate cancers from exhaled breath using a single array of nanosensors. *Br. J. Cancer* **2010,** *103*, 542-551.
2. Peng, G.; Tisch, U.; Adams, O.; Hakim, M.; Shehada, N.; Broza, Y. Y.; Billan, S.; Abdah-Bortnyak, R.; Kuten, A.; Haick, H., Diagnosing lung cancer in exhaled breath using gold nanoparticles. *Nature Nanotechnol.* **2009,** *4*, 669-673.
3. Helmig, D.; Vierling, L., Water adsorption capacity of the solid adsorbents Tenax TA, Tenax GR, Carbotrap, Carbotrap C, Carbosieve SIII, and Carboxen 569 and water management techniques for the atmospheric sampling of volatile organic trace gases. *Anal. Chem.* **1995,** *67*, 4380-4386.
4. Broza, Y. Y.; Haick, H., Nanomaterial-based sensors for detection of disease by volatile organic compounds. *Nanomedicine (Future Medicine)* **2013,** *8*, 785-806.
5. Konvalina, G.; Haick, H., Effect of humidity on nanoparticle-based chemiresistors: a comparison between synthetic and real-world samples. *ACS. Appl. Mater. Interf.* **2011,** *4*, 317-325.
6. Konvalina, G.; Haick, H., Sensors for breath testing: from nanomaterials to comprehensive disease detection. *Acc. Chem. Res.* **2014,** *47*, 66-76.
7. Tisch, U.; Haick, H., *Sensors based on monolayer-capped metal nanoparticles*. Momentum Press: LLC, New York, Vol. 2, (Nanstructured Materials): 2010; Vol. Chapter 4, p 141-202.
8. Amann, A.; Spanel, P.; Smith, D., Breath analysis: the approach towards clinical applications. *Mini-Rev. Med. Chem.* **2007,** *7*, 115-129.
9. Amann, A.; Miekisch, W.; Pleil, J.; Risby, T.; and Schubert, J., Methodological issues of sample collection and analysis of exhaled breath. In *Eur. Resp. Soc. Monogr.*, 2010; Vol. 49, pp 96–114.
10. Hakim, M.; Broza, Y. Y.; Barash, O.; Peled, N.; Phillips, M.; Amann, A.; Haick, H., Volatile organic compounds of lung cancer and possible biochemical pathways. *Chem. Rev.* **2012,** *112*, 5949-5966.
11. Filipiak, W.; Sponring, A.; Filipiak, A.; Ager, C.; Schubert, J.; Miekisch, W.; Amann, A.; and Troppmair, A., TD-GC-MS analysis of volatile metabolites of human lung cancer and normal cells in vitro. *Cancer. Epidemiol. Biomarkers. Prev.* **2010,** *19*, 182-195.
12. Tisch, U.; Haick, H., Nanomaterials for cross-reactive sensor arrays. *Bull. MRS* **2010,** *35*, 797-803.
13. Dovgolevsky, E.; Haick, H., Direct observation of the transition point between quasi-spherical and cubic nanoparticles in a two-step seed-mediated growth method. *Small* **2008,** *4*, 2059-2066.
14. Dovgolevsky, E.; Tisch, U.; Haick, H., Chemically sensitive resistors based on monolayer-capped cubic nanoparticles: towards configurable nanoporous sensors. *Small* **2009,** *5*, 1158-1161.
15. Dovgolevsky, E.; Konvalina, G.; Tisch, U.; Haick, H., Monolayer-capped cubic platinum nanoparticles for sensing nonpolar analytes in highly humid atmospheres. *J. Phys. Chem. C.* **2010,** *114*, 14042-14049.
16. Zilberman, Y.; Ionescu, R.; Feng, X.; Müllen, K.; Haick, H., Nanoarray of polycyclic aromatic hydrocarbons and carbon nanotubes for accurate and predictive detection in real-world environmental humidity. *ACS Nano* **2011,** *5*, 6743-6753.
17. Zilberman, Y.; Tisch, U.; Pisula, W.; Feng, X.; Müllen, K.; Haick, H., Spongelike structures of hexa-peri-hexabenzocoronene derivatives enhance the sensitivity of chemiresistive carbon nanotubes to nonpolar volatile organic compounds of cancer. *Langmuir* **2009,** *25*, 5411-5416.
18. Peng, G.; Trock, E.; Haick, H., Detecting simulated patterns of lung cancer biomarkers by random network of single-walled carbon nanotubes coated with nonpolymeric organic materials. *Nano Lett.* **2008,** *8*, 3631-3635.
